# Incidence and prevalence of rabies virus infections in tested humans and animals in Asia: A systematic review and meta-analysis study

**DOI:** 10.1016/j.onehlt.2025.101102

**Published:** 2025-06-05

**Authors:** Farzane Shams, Mohammad Jokar, Ehssan Djalali, Arman Abdous, Mehdi Rahnama, Vahid Rahmanian, Kaushi S.T. Kanankege, Torsten Seuberlich

**Affiliations:** a Division of Neurological Sciences, Vetsuisse Faculty, Universitoy of Bern, Bern, Switzerland; b Faculty of Veterinary Medicine, University of Calgary, Calgary, AB T2N 1N4, Canada; c Faculty of Veterinary Medicine, Karaj Branch, Islamic Azad University, Karaj, Iran; d Young Researchers and Elites Club, Karaj Branch, Islamic Azad University, Karaj, Iran; e Faculty of Veterinary Medicine, Science and Research Branch, Islamic Azad University, Tehran, Iran; f Department of Public Health, Torbat Jam Faculty of Medical Sciences, Torbat Jam, Iran; g College of Veterinary Medicine, University of Minnesota, USA; h Graduate School for Cellular and Biomedical Sciences, University of Bern, Bern, Switzerland

**Keywords:** *Rhabdoviridae*, *Lyssavirus*, Rabies, Dog, Asia, Prevalence, Incidence

## Abstract

**Introduction:**

Rabies is a fatal neurological zoonotic disease affecting warm-blooded animals, causing nearly 60,000 human deaths annually, primarily in developing Asian and African countries (95 % of cases). This review examines the prevalence and incidence of rabies in tested humans and animals across Asia.

**Methods:**

We searched for scientific articles published in peer-reviewed journals between 2010 and 2024 in electronic databases. Ninety-seven publications were selected for the assessment of the rabies prevalence and nine for the assessment of the rabies incidence.

**Results:**

Overall, the prevalence of rabies based on the random-effects meta-analysis was 23 % (95 % CI 22.7–23.4) in tested animals and 52 % (95 % CI 40.2–63.8) in tested humans. Among animals, foxes had the highest test prevalence of 78.3 % (95 % CI 70.4 %–86.2 %) followed by dogs (38.1 %, 95 % CI 31.2 %–45 %). The incidence in tested animals was 0.5 % (95 % CI 0.4 %–0.6 %) and 0 % (95 % CI 0 %–0 %) in tested humans. Among animals, dogs have the highest incidence at 0.7 % (95 % CI 0.5 %–0.8 %).

**Conclusion:**

Many Asian countries have eradicated rabies by implementing control measures such as animal registration, quarantine, isolation, and mandatory mass vaccination. However, rising fox populations now pose a potential risk for rabies spread in the region.

## Highlights

• Rabies remains a major health issue in Asia, with prevalences of 23 % in tested animals and 52 % in tested humans.
• Foxes show the highest prevalence of rabies in tested animals (78.3 %), highlighting the need for wildlife monitoring.
• Differences in the eradication strategies shape rabies conditions across Asian countries.

## 1 Introduction

Rabies virus (RABV) is a member of the genus *Lyssavirus* within the family *Rhabdoviridae* and causes rabies, a neurological zoonotic disease, infects domestic and wild warm-blooded animals [1,2]. Infected animals can transmit the virus through biting, scratching, or licking open wounds. After transmission, the virus travels via nerves to the brain, causing neurological symptoms, and then replicates in the salivary glands resulting in high virus excretion. While various animals can transmit RABV, domestic dogs cause over 99 % of human rabies deaths in developing countries [2]. RABV is among the deadliest pathogens, causing approximately 60,000 deaths worldwide with an estimated economic burden of $8.6 billion annually, except for Antarctica and Australia [3].

There are several factors resulting in underreporting of rabies in Asia, which include misinformation about the rabies virus and reservoir hosts among the public and healthcare workers, lack of reliable diagnostic tests, absence of postmortem examination in particular in animals with signs if bites, lack of reports on affected and/or killed animals because of culture practices and insufficient monitoring of wildlife and human contact [4,5].

There is considerable variation of economic power among Asian countries, with 78 % falling into low and middle-income categories, reflecting significant economic challenges. In contrast, countries like Qatar, Japan, Israel, Kuwait, and others are among those with the highest income [6]. Additionally, while some countries are in a stable political situation, others face significant political challenges that impact also on the public health sector. In these contexts, priorities often shift from endemic diseases like rabies to more immediate concerns such as infectious disease outbreaks, malnutrition, or maternal and child health, further exacerbating the rabies burden [7]. Despite rabies being a 100 % fatal disease, it is preventable through post-exposure prophylaxis (PEP) and vaccination. However, the costs of these measures are prohibitive for many countries, limiting their effectiveness in preventing disease [7,8].

Rabies is predominantly diagnosed in Asia and Africa, accounting for 95 % of positive cases in the world [6]. Underreporting and limited diagnostics lead to an underestimation of rabies' true burden, especially in developing countries [9]. Rabies is endemic in 24 of the 49 countries in Asia, and given the high number of human victims, a systematic analysis of the current epidemiological landscape in Asia is urgently needed. To this end, we conducted a study examining rabies incidence and prevalence based on reported tests to improve our understanding of RABV spread in animals and humans across Asia. By consolidating data from various studies on rabies among animal populations of different species and human populations in Asian countries, this study provides the first systematic review and meta-analysis of RABV infection incidence and prevalence in tested animals and tested humans from 2010 to 2024. These insights are crucial for identifying surveillance gaps in one of the world's rabies-endemic regions and align with WHO's disease eradication goals.

## 2 Materials and methods

### 2.1 Study design

The current study was conducted according to the “preferred reporting for systematic reviews and meta-analyses” (PRISMA) guidelines [10]. We defined the inclusion criteria based on the Joanna Briggs recommendation, which is used for systematic reviews, incidence and prevalence. A country was defined to belong to Asia based on the National Geographic Society's classification (https://www.nationalgeographic.org/resource/asia-human/). This analysis considered all population-level studies involving relevant animal species and humans that were tested or reported to have rabies. Patients were classified into healthy and rabid cases. All samples, including the brain, serum, saliva, cerebrospinal fluid, and any tissue biopsy, were considered in this study. In addition, all types of valid diagnostic methods including molecular, immune-enzymatic, immunochromatographic, immunofluorescent, histopathological, and culture-based assays, were considered. We excluded case reports, reviews, commentaries, studies involving experimental RABV infections, duplicates, and studies for which only abstracts were available. Studies with a sample size of fewer than 10 were excluded for animal hosts; however, due to the importance of human rabies, studies with fewer than 10 human cases were included in the analysis.

### 2.2 Search strategy

To better understand the epidemiology of rabies in Asia, we examined both prevalence and incidence data. Prevalence reflects the proportion of individual animals or humans infected with rabies at a specific time or over a period, offering insight into the existing disease burden within a population. In contrast, incidence measures the number of new cases occurring during a particular time and shows the disease emergence or transmission rate. Considering both metrics provides a more complete understanding of rabies dynamics: prevalence informs on endemicity and surveillance coverage, while incidence reveals patterns of spread and potential outbreaks.

Articles which were published between January 2010 and 2024 were selected from eight databases, i.e., Google Scholar, Pub Med, Scopus, Embase, Web of Science, Science Direct, ProQuest, and Springer. Only studies published in English were selected for the initial review process. The authors conducted a comprehensive search using Medical Subject Headings (MeSH) terms including “Rabies,” “RABV,” “Epidemiological survey,” “Prevalence”, “Seroprevalence,” “Incidence”, “Animal”, “Human”, along with the names of individual Asian countries for English-language sources. Additionally, all related articles recommended in the databases were reviewed. The study selection process is illustrated in the PRISMA flowchart (Supplementary Fig. 1).

### 2.3 Selection and inclusion criteria

Studies were screened by title, abstract, and full text by two authors. Studies were then assessed against predefined exclusion criteria (Supplementary Fig. 1). Discrepancies were resolved by a third author. Key data extracted included author, year, country, sample size, RABV cases, species, bias risk, and diagnostic methods including direct fluorescent antibody test (DFA), reverse transcription polymerase chain reaction (RT-PCR), histology, fluorescent antibody virus neutralization (FAVN), neutralizing peroxidase-linked assay (NPLA), Seller's staining and magnetic resonance imaging (MRI).

### 2.4 Quality assessment

We evaluated the quality of studies that met the inclusion criteria using the Newcastle–Ottawa scale (NOS), a widely recognized method for systematic reviews and meta-analyses as a tool for assessing the quality of non-randomized studies that grades studies based on three key domains: selection of study groups, comparability of groups, and ascertainment of either exposure or outcome of interest [11]. The NOS assigns scores to studies, with a maximum score of 9 and are classified as high quality (>6), moderate quality [[3], [4], [5]], and low quality (<3) [12].

### 2.5 Statistical analysis

The random-effects model was used to calculate pooled estimates of overall incidence, prevalence and 95 % confidence intervals (CI) [13]. For both incidence and prevalence, the 95 % confidence interval provides a range within which the true population parameter (incidence rate or prevalence) is likely to fall. Moreover, Cochran's Q test, and the I2 index were used to evaluate heterogeneity among the studies using Stata 14 (https://www.stata.com/stata14/). The number of individual studies and overall incidence, prevalence, and heterogeneity were demonstrated through forest plots. Multivariable and univariable meta-regression methods and subgroup analyses were also used to calculate the effects of the eventual factors on heterogeneity. Subgroup analysis allows for the examination of differences in outcomes between various subgroups within the study population, providing deeper insights into how different factors or characteristics may influence the results or effectiveness of interventions.

Subgroup analyses were conducted by country, diagnostic method, and animal species to explore sources of heterogeneity and factors influencing rabies prevalence estimates. The publication bias was assessed by funnel plotting and Egger's regression test. The ArcGIS 10.3 software (https://www.arcgis.com) was utilized to visualize the incidence and prevalence of RABV in animals and humans in the different Asian countries. The meta-analysis was performed using the trial version of the Stat Direct statistical software (https://www.statsdirect.com) [14].

## 3 Results

### 3.1 Study selection and characteristics of eligible studies

A total of 2173 studies were identified by the initial search. After deduplication, 1197 articles remained. Among these, 574 articles met our inclusion criteria based on their titles and abstracts. Ultimately, we selected 97 articles reporting on rabies test positive prevalence and 9 articles on rabies test positive incidence (Supplementary Fig. 1). As per the NOS criteria, for prevalence, 63 studies were considered as high quality and 34 of medium quality; and for incidence 9 studies were considered as high quality and 0 of medium quality.

### 3.2 Baseline characteristics of considered studies

Eligible studies covered 26 out of 49 (53 %) Asian countries that published population-level rabies studies between 2010 and 2024, including testing of 13 different animal species and humans (Supplementary tables 1, 2, 3 and 4). Most of the animal studies were from China (*n* = 18) and India (*n* = 9). Of the diagnostic tests, DFA was most used for detecting RABV in suspected samples (60.9 % among the studies). It should be noted that 5 studies reported pooled RABV incidence data for various animals without specifying the species. Furthermore, two studies on human patients and two studies on animals did not report on the diagnostic methods used (Table 1, Table 2, Table 3).

**Table 1:** Pooled prevalence of rabies in tested animals.

		No. studies	No. examined	No. positive	Prevalence (95 %CI)	Heterogeneity		
						Q	P-value	I^2^ (%)
Country	China	18	26,401	1237	14.5 [12.2 %–16.8 %]	2366.99	<0.01	99 %
	Jordan	3	52	34	66.2 [53.5 %–78.9 %]	1.47	0.480	0 %
	Iraq	2	98	56	49.5 [0 %–100 %]	227.50	<0.01	99.5 %
	Philippines	6	1995	855	37.1 [19.3 %–54.9 %]	735.24	<0.01	98.6 %
	Seri Lanka	6	21,226	11,246	52 [38.2 %–65.9 %]	1985.00	<0.01	99.7 %
	Indonesia	2	12,788	3747	45.7 [0 %–100 %]	6794.59	<0.01	99.9 %
	India	9	1562	812	60.6 [50.6 %–70.6 %]	177.87	<0.01	93.3 %
	South Korea	4	1237	488	34.3 [18 %–50.6 %]	102.29	<0.01	97 %
	Taiwan	3	1634	836	50.3 [20.1 %–80.4 %]	256.74	<0.01	98.2 %
	Vietnam	3	1841	217	11.7 [0 %–27.9 %]	242.81	<0.01	99.2 %
	Oman	3	1114	620	58.7 [51.8 %–65.7 %]	17.48	<0.01	84.5 %
	Georgia	2	4367	949	33.4 [7.7 %–59.2 %]	10.15	<0.01	90.1 %
	Iran	5	11,694	3414	65.8 [62.5 %–69.1 %]	49.77	<0.01	77.9 %
	Mongolia	3	152	119	80.5 [66.2 %–94.8 %]	18.58	<0.01	94.6 %
	Nepal	2	3107	1516	61.6 [50.4 %–72.7 %]	26.82	<0.01	85.1 %
	Saudi Arabia	2	230	188	83.2 [76.6 %–89.7 %]	11.30	0.080	46.9 %
	Thailand	4	28,946	3382	21.8 [15.5 %–28 %]	1039.40	<0.01	99.4 %
	Turkey	1	881	340	39.2 [29.1 %–49.3 %]	30.39	<0.01	90.1 %
	Yemen	1	166	104	62.8 [55.4 %–70.1 %]	NA	NA	NA
	Lao PDR	2	1664	924	59.7 [42.9 %–76.6 %]	41.17	<0.01	97.6 %
	Pakistan	2	90	9	29.8 [0 %–84.8 %]	14.42	<0.01	93.1 %
	Tajikistan	1	59	7	12.6 [4.2 %–21 %]	NA	NA	NA
	Azerbaijan	1	326	216	66.3 [61.2 %–71.4 %]	NA	NA	NA
	Bangladesh	2	4,728,407	14,145	0.3 [0.2 %–0.3 %]	694.84	<0.01	99.4 %
Detection method	DFA	60	84,065	23,133	48.4 [44.3 %–52.5 %]	54,638.47	<0.01	99.8 %
	FAVN	1	94	33	35.4 [25.8 %–45.1 %]	NA	NA	NA
	Histological	2	120	40	48.1 [0 %–100 %]	360.39	<0.01	99.7 %
	NPLA	1	533	288	54.1 [49.8 %–58.3 %]	NA	NA	NA
	RT-PCR	12	7048	1339	37.2 [26.8 %–47.5 %]	2650.90	<0.01	99.6 %
	Seller's staining	1	169	125	74 [67.4 %–80.6 %]	NA	NA	NA
	UK	10	4,758,008	24,503	10.2 [9.8 %–10.5 %]	24,572.23	<0.01	99.9 %
Species	Ferret badger	7	2740	634	20.8 [1.7 %–39.9 %]	1595.89	<0.01	99.6 %
	Dog	33	51,222	14,689	38.1 % [31.2 %–45 %]	37,658.09	<0.01	99.9 %
	Bat	5	4868	294	6.8 [2.2 %–11.4 %]	248.90	<0.01	98.4 %
	Cat	7	2833	591	21.3 [13 %–29.6 %]	52.01	<0.01	90.4 %
	Raccoon dog	2	109	38	34.9 [25.9 %–43.8 %]	NA	NA	NA
	Buffalo	3	139,901	230	38.3 [0 %–88.4 %]	263.01	<0.01	99.2 %
	Cow	10	2,901,892	9679	58.7 [29.9 %–87.4 %]	3016.43	<0.01	99.7 %
	Horse	1	11	5	47.8 % [19 %–76.7 %]	NA	NA	NA
	Sheep	6	142,017	552	47.8 [16.6 %–79.1 %]	377.62	<0.01	98.9 %
	Goat	3	1,545,875	4540	56.3 % [0 %–100 %]	323.01	<0.01	99.4 %
	Camel	2	107	74	72.7 [48 %–97.4 %]	9.51	<0.01	89.5 %
	Fox	4	104	81	78.3 [70.4 %–86.2 %]	0.03	0.866	0 %
	Mouse	1	45	1	3.3 [0 %–8.5 %]	NA	NA	NA

**Table 2:** Pooled prevalence of rabies in tested humans.

		No. studies	No. examined	No. Positive	Prevalence[ 95 %CI]	Heterogeneity		
						Q	P-value	I^2^ (%)
Country	Iran	2	67	46	68.7 [57.6 %–79.8 %]	0.09	0.764	0 %
	Seri Lanka	2	581	456	81.7 [71.7–91.7]	54.08	<0.01	74.1 %
	China	3	3321	486	37 [9.7 %–64.3 %]	83.28	<0.01	97.6 %
	Iraq	1	732	409	55.9 [52.3 %–59.5 %]	NA	NA	NA
	Vietnam	1	31	23	74.6 [59.4 %–89.8 %]	NA	NA	NA
	Malaysia	1	6	5	84.6 [56.9 %–100 %]	NA	NA	NA
	India	2	122,557	147	20.1 [0 %–59.8 %]	87.24	<0.01	98.9 %
	Yemen	1	76,049	21,927	28.8 [28.5 %–29.2 %]	NA	NA	NA
Detection method	DFA	6	3928	846	59.8 [28.8 %–90.7 %]	59.69	<0.01	93.3 %
	MRI	1	502	387	77.1 [73.4 %–80.8 %]	52.50	<0.01	75.2 %
	RT-PCR	4	436	244	55.5 [38.7 %–72.3 %]	24.28	<0.01	87.6 %
	UK	2	198,478	22,022	14.5 [0 %–42.6 %]	30,584.89	<0.01	100 %

**Table 3:** Pooled incidence of rabies in tested animals.

		No. studies	No. examined	No. positive	Prevalence (95 %CI)	Heterogeneity		
						Q	P-value	I^2^ (%)
Country	Bhutan	2	109,021	350	0.5 [0.3 %–0.7 %]	140.82	<0.01	96.4 %
	India	1	8,100,000	30	0 [0 %–0 %]	NA	NA	NA
	Oman	1	22,722	425	1.9 [1.7 %–2 %]	NA	NA	NA
	Iran	1	105,896	62	0.1 [0 %–0.1 %]	NA	NA	NA
Detection method	DFA	1	8,100,000	30	0 [0 %–0 %]	NA	NA	NA
	RT-PCR	2	128,618	487	1 [0 %–2.7 %]	403.65	<0.01	99.8 %
	Unknown	2	109,021	350	0.5 [0.3 %–0.7 %]	140.82	<0.01	96.4 %
Species	Dog	5	8,242,430	675	0.7 % [0.5 %–0.8 %]	654.13	<0.01	99.4 %
	Cat	1	1156	2	0.2 [0 %–0.4 %]	NA	NA	NA
	Cow	2	85,696	183	0.3 [0 %–0.7 %]	17.83	<0.01	94.4 %
	Horse	1	8357	7	0.1 % [0 %–0.1 %]	NA	NA	NA

### 3.3 Pooled prevalence of rabies infection in animals and humans

In total, test results for 4,848,734 animals and 203,237 humans were reported in the studies included. The diagnostic results showed that 49,413 animals and 23,400 humans tested positive. The overall prevalence of rabies, based on a random-effects meta-analysis among the tested animals was 23 % (95 % CI 22.7–23.4) and 52 % (95 % CI 40.2–63.8) among tested humans (Supplementary Fig. 2).

The heterogeneity degree between the different studies estimating the rabies prevalence in tested animals was high: Q statistic = 88,424.42 (df = 125), *P* < 0.0001, and I 2 = 99.9 %. Similarly, a high degree of heterogeneity was observed for tested humans: Q statistic = 34,551.82 (df = 12), P < 0.0001, and I 2 = 100 %.

### 3.4 Pooled incidence of rabies infection in animals and humans

There were 8,337,639 animals and 163,727,530 humans considered for the analysis of RABV incidence. The results showed that 867 animals and 3020 humans tested positive for RABV. Based on the random-effects meta-analysis, the overall incidence of rabies among animals was 0.5 % (95 % CI 0.4–0.6) and among humans 0 % (95 % CI 0–0) (Supplementary Figs. 3 and 4). The degree of heterogeneity between studies that reported RABV incidence among animals was high: Q statistic = 845.90 (df = 8), *P* = 0.0001, and *I* = 99.1 %. Likewise, a high degree of heterogeneity was observed between the different studies estimating rabies incidence among humans: Q statistic = 2769.06 (df = 10), P = 0.0001, and *I* = 99.6 %.

### 3.5 Factors determined by sub-unit analysis

#### 3.5.1 Prevalence

##### 3.5.1.1 Animals

**Fig. 1:**
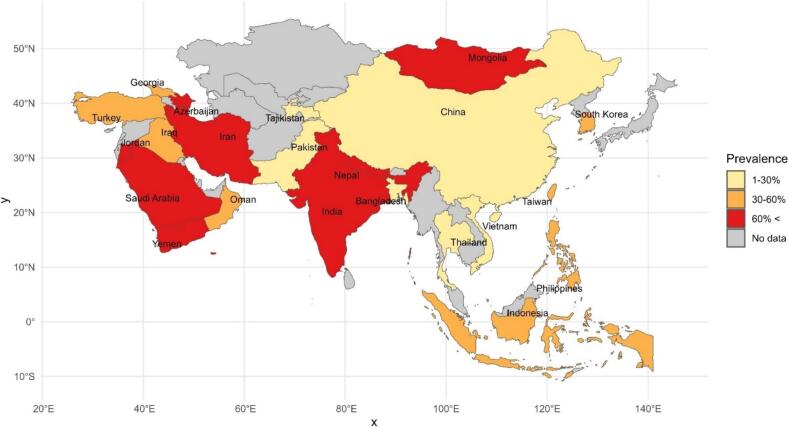
Prevalence of rabies in tested animals in Asian countries (2010–2024).

The results of total prevalence based on the diagnosis methods applied were as follows: RT-PCR 37.2 % (95 % CI 26.8–47.5), DFA 48.4 % (95 % CI 44.3–52.5), NPLA 54.1 % (95 % CI), FAVN 35.4 % (95 % CI 25.8–45.1), Seller's staining 74 % (95 % CI), and histology 48.1 % (95 % CI 0–100) (Table1). The highest and lowest prevalence among the countries were for Saudi Arabia 83.2 % (95 %CI 76.6–89.7) and Bangladesh 0.3 % (95 % CI 0.2–0.3), respectively (Table and Fig. 1). According to the animal species, the highest was in foxes at 78.3 % (95 % CI 70.4–86.2) followed by camels at 72.7 % (95 % CI 48–97.4), and the lowest was in mice at 3.3 % (95 % CI 0–8.5) (Table 1).

The univariate meta-regression analysis did not show heterogeneity in diagnostic methods (*p* = 0.423), country (*p* = 0.986), and animal species (*p* = 0.394). Furthermore, the multivariate meta-regression test revealed no significant differences between diagnostic methods (*p* = 0.56), country (*p* = 0.089), and animal species (*p* = 0.131). At *p*-values 0.089 in univariate analysis and 0.06 in multivariate analysis, the country variable indicated marginal significance contributing to explain variability in the effect sizes across studies with focus on detecting animal rabies.

##### 3.5.1.2 Humans

**Fig. 2:**
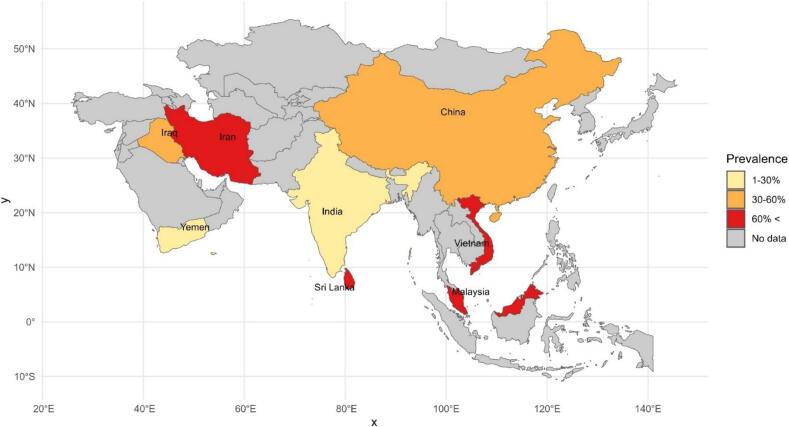
Prevalence of rabies in tested humans in Asian countries (2010–2024).

The results of total prevalence based on the diagnosis methods have been estimated for DFA 59.8 % (95 % CI 28.8–90.7), RT-PCR 55.5 % (95 % CI 38.7–72.3), MRI 77.1 % (95 % CI 73.4–80.8) (Table 2). The highest and lowest prevalence among the countries was for Malaysia at 84.6 % (95 %CI 56.9–100) and the lowest was for India at 20.1 % (95 % CI 0–59.8). A schematic map of rabies case distribution in humans was designed based on studies analyzed in Asia and countries globally (Fig. 2).

The multivariate meta-regression test did not demonstrate heterogeneity in diagnostic method (*p* = 0.144) and country (*p* = 0.079). Furthermore, the univariate meta-regression did not show heterogeneity in the country (*p* = 0.150) and diagnostic methods (*p* = 0.524). With a *p*-value of 0.079 in the multivariate analysis, after adjusting for multiple moderators, the country variable showed marginal significance in explaining the variability in effect sizes across studies on human rabies diagnostics.

#### 3.5.2 Incidence

**Fig. 3:**
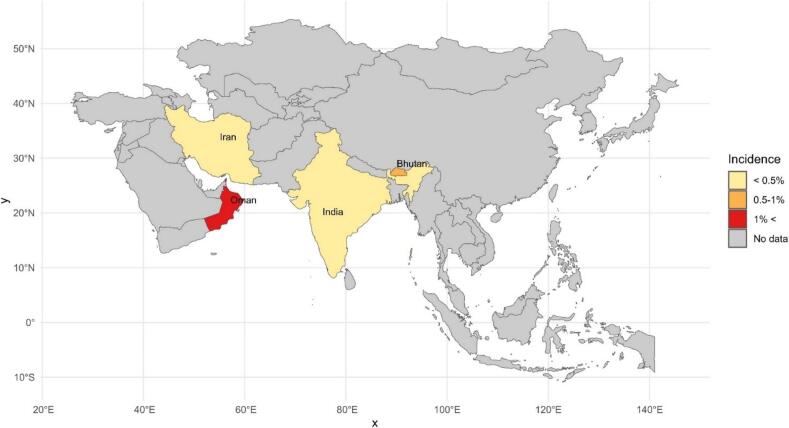
Incidence of rabies in tested animals in Asian countries.

Based on the diagnosis methods, the total incidence results are as follows: 1 % (95 % CI 0–2.7) for RT-PCR and 0 % for DFA (Table 3). Moreover, the incidence rates vary between countries. Oman had the highest incidence rate of 1.9 % (95 %CI 1.7–2), while India had a incidence rate of 0 (Table 3). A Schematic map was designed to show the global RABV incidence rates in animals (Fig. 3). The highest incidence rate was found in dogs with 0.7 % (95 %CI 0.5–0.8), while the lowest was found in mice with 0.1 % (95 % CI 0.01–0.102) (Table 3).

The univariate meta-regression analysis showed that none of the studied variables significantly contributed to explaining the heterogeneity in effect sizes: diagnostic methods (*p* = 0.745), country (*p* = 0.986), and animal species (*p* = 0.225). Furthermore, the multivariate meta-regression test showed that there were no significant differences in diagnostic methods (*p* = 0.322), country (*p* = 0.27), and animal species (*p* = 0.281).

#### 3.5.3 Publication bias

We applied the funnel plot and Egger's test to identify publication bias. The result of Egger's test for the studies reporting the prevalence of rabies in tested animals showed a bias of 16.3 (95 % CI 12.53–20.07, *P* < 0.001) and for tested humans, the bias was 28.23 (95 % CI -2.34-58.8, *P* = 0.067). Additionally, Egger's test revealed a bias of 8.6 (95 % CI 3.52–13.67, *P* = 0.005) for studies reporting the incidence of rabies in animals and a bias of 21.89 (95 % CI 6.57–37.21, *P* = 0.01) in humans.

Therefore, the asymmetry in the funnel plot interpretation and the results of Egger's test indicate significant publication bias in studies reporting the rabies prevalence in tested animals as well as the rabies incidence in animals and humans (Supplementary figs. 5 and 6).

## 4 Discussion

Rabies is one of the most important zoonotic diseases and endemic in most regions of Asia. This systematic review and meta-analysis study aimed to provide reliable combined data on the prevalence of RABV among tested 203,237 humans (52 %) and 4,848,734 tested animals (23 %) in Asia and on the incidence in 163,727,530 tested humans (0 %) and in 8,337,639 tested animals (0.5 %). According to the world health organization (WHO) report 95 % of rabies cases occur in Asia and Africa [15]. A study reported the rabies prevalence of 83.4 % in tested humans and 41.4 % in tested animals in Africa, which is higher than in Asia. Low vaccination rates (16 % in Asia, 14 % in Africa) may contribute to this difference. Vaccinating >70 % of dogs can eliminate transmission and reduce human risk [16]. However, the reported number of laboratory-confirmed rabies cases is limited for both human and animal populations; and likely underestimates the true impact of this neglected zoonotic disease, especially in enzootic and epizootic areas of Asia and Africa [17,18]. The study also indicates that the country is a marginally influential factor determining the heterogeneity of rabies prevalence and incidence diagnostics.

Although diagnostic prevalence and incidence in tested humans and animals may not perfectly reflect the true burden of rabies due to the given differences in testing practices, case definitions, and surveillance infrastructure, both parameters are valuable for identifying trends and regional risk. Together, these measures offer insight into both current disease load and transmission dynamics across different settings.

Although diagnostic prevalence and incidence may not perfectly reflect the true burden of rabies due to the given differences in testing practices, case definitions, and surveillance infrastructure. They remain valuable for identifying trends and regional risk. Together, these measures offer insight into both current disease load and transmission dynamics across different settings.

In our analysis, Western Asia (including Jordan, Iraq, Oman, Georgia, Saudi Arabia, Turkey, Yemen, and Azerbaijan] showed the highest diagnostic prevalence of rabies in animals. This may reflect a combination of endemic transmission and decreasing vaccination coverages, increased interactions between wildlife and domestic animals, and relatively better diagnostic reporting in some countries. On the other hand, Southern Asian countries reported more suspected cases but a lower prevalence, which could be correlated to stronger public health awareness, broader implementation of animal vaccination programs, and increased access to post-exposure prophylaxis (PEP) [16,19]. These findings highlight the critical role of targeted interventions in modifying disease prevalence, especially in endemic regions.

In Malaysia, the diagnostic prevalence of rabies in tested human cases was remarkably high (84.6 %), particularly in Sarawak on Borneo Island, a region bordering known rabies-endemic zones [20]. Similarly, Yemen and Sri Lanka reported the highest absolute numbers of human rabies cases, underscoring the status of rabies as a neglected zoonotic disease in parts of the region. Contributing factors include limited public awareness, low animal vaccination coverage, presence of wildlife reservoirs, and variations in detection methods [15].

Saudi Arabia recorded the highest animal rabies prevalence (83.4 %), with most cases involving camels, sheep, and goats. The proximity of cities to desert ecosystems likely increases contact between wild carnivores and farm animals or humans. Additionally, rabies remains a public health concern in domestic pets such as dogs and cats [21]. Oman showed the highest incidence rate in animals, likely driven by rabies circulation in wildlife, particularly foxes, and their contact with stray dogs and cats [22]. On the other end of the spectrum, Bangladesh reported the lowest prevalence in animals (0.3 %). Several reports have highlighted the limited vaccination coverage in animals in the country, which increases the risk of rabies transmission [23,24]. Therefore, these numbers likely reflect underreporting rather than an actual low infection rate.

The DFA is the gold standard for rabies diagnosis, with 100 % sensitivity, approved by WHO and WOAH for fresh brain tissue testing. However, it poses exposure risks and reduced sensitivity if samples are autolyzed or improperly fixed [5]. RT-PCR, recently confirmed by WOAH, is a molecular diagnostic alternative for post-mortem, ante-mortem, and sub-optimal samples, as it does not require fresh tissue or live viruses [25]. Histological methods such as Seller's stain are not recommended because of the low sensitivity [5]. MRI is not a routine test; however, it can be useful in cases with a less obvious clinical picture [26]. Choosing biological samples plays a major role in the detection of RABV [21].

Previous studies had indicated that the number of fox rabies in the Middle East and Central Asia had been increasing during the years 2013–2022 [1,21]. Our results indicate foxes may drive rabies in Asia, with the highest diagnostic prevalence (78.3 %). It should be noted that rabies is not a common disease in rats, although a bite from them should not be ignored [27]. Incidence data show cows as the most at-risk dead-end hosts, likely due to farm proximity to wildlife and dogs. In China, livestock are often rabies infected by bites of infected foxes and raccoon dogs, thus a licensed vaccine for farm animals is recommended in high-risk regions [1,28].

Humans are mostly infected by cat and dog bites or scratches. It should be noted that rabies is not transmitted through intact skin (WHO). Additionally, RABV is not an airborne virus; however, in some exceptional circumstances, such as in a caves with important bat populations, transmission may occur via aerosols [29]. WHO data for the years 2010–2021, show two rabies waves: a peak in 2010–2011, a decline, and a smaller 2016–2017 resurgence (Supplementary Fig. 7). China had the highest case numbers, while Thailand, Vietnam, and the Philippines reported steady numbers. Cases dropped sharply after 2018, nearing zero by 2021, likely due to restrictions during the COVID-19 pandemic. Despite vaccination being the most effective protective measure, some rabies endemic countries, like China, have no vaccination guidelines for cats; they prefer to control rabies in dogs as the main reservoir to reduce RABV in cats [30]. On the other hand, some Asian countries such as Japan, Singapore, Kuwait, Qatar, Israel, Bahrain, Armenia, and US Emirates have eliminated dog-mediated rabies for many years and continue to keep the status by animal registration, quarantine, mandatory vaccination of animals [31], implementation of rigorous importation rules for pets, and by elimination of stray dogs [32]. In contrast, counties including Timor-Leste, Cambodia, Syria, North Korea, Brunei, Palestine, Maldives, Myanmar, Kyrgyzstan, Lebanon, Vietnam, Afghanistan, Cyprus, Uzbekistan, Kazakhstan, Turkmenistan and Macao have no significant studies and are not represented in WHO reports on rabies.

Previous scientific review articles from various Asian countries address the gaps and the necessity of having an overall rabies prevalence assessment in Asia [33,34]. Addressing this gap is important for improving disease detection and understanding its impact on these populations. Prevalence and incidence rates play a key role in evaluating how effectively rabies is identified, informing public health actions such as vaccination programs and resource allocation. Moreover, these metrics are key numbers to assess the disease situation, to implement timely disease control actions, and to eventually reduce mortality rates.

### 4.1 Limitations

Our study has several limitations. We did not consider vaccination status, eradication programs and seroprevalence rates due to limited available data sources. We also excluded studies that reported positive cases while omitting important data such as total suspected cases or populations at risk. Additionally, some countries focused more on rabies disease control and consequently more data are available, compared to other endemic countries. Besides, we faced information gaps from some Asian countries due to political issues.

## 5 Conclusion

Rabies continues to be a public health threat across Asia, with high prevalence in both animals and humans, indicating gaps in disease control strategies. While the overall numbers are alarming, the situation varies significantly between countries due to differences in wildlife ecology, urbanization, animal vaccination coverage, and public health infrastructure.

In countries like Saudi Arabia and Oman, the interaction between wild animals and livestock, especially in areas near deserts, drives much of the rabies burden, suggesting a need for physical barriers and wildlife monitoring around farms. In contrast, rabies-endemic countries such as India and China face persistent challenges related to dog-mediated transmission, underscoring the need for stronger regulations on vaccination, effective population control measures for stray dogs, and widespread public awareness campaigns. Meanwhile, countries in Southeast Asia are more affected by cross-border transmission and wildlife reservoirs, emphasizing the importance of regional coordination and border surveillance.

These findings underscore that rabies control can't rely on one single strategy and demands for a One Health approach, i.e. the integration of the human, animal, and environmental health sectors to share data, design joint interventions, and build sustainable systems for prevention. Without concerted action, rabies will remain a neglected disease despite being entirely preventable.

## CRediT authorship contribution statement

**Farzane Shams:** Writing – original draft, Validation, Investigation, Data curation, Conceptualization. **Mohammad Jokar:** Formal analysis, Data curation, Conceptualization. **Ehssan Djalali:** Visualization, Validation. **Arman Abdous:** Visualization, Validation, Formal analysis, Data curation. **Mehdi Rahnama:** Validation, Data curation. **Vahid Rahmanian:** Formal analysis. **Kaushi S.T. Kanankege:** Writing – review & editing, Writing – original draft, Supervision, Conceptualization. **Torsten Seuberlich:** Writing – review & editing, Writing – original draft, Supervision, Conceptualization.

## Funding

This study did not involve external funding.

## Declaration of competing interest

The authors declare no competing interests.

## Data Availability

All papers in this study are properly cited. For any questions regarding the analysis, readers may contact the corresponding or first authors via email.

## Appendix A Supplementary data

Supplementary material Image 1
