# Supplementary material for: Incidence and prevalence of rabies virus infections in tested humans and animals in Asia: A systematic review and meta-analysis study
Source: One Health. 2025 Jun 5;20:101102. doi: 10.1016/j.onehlt.2025.101102 (PMC12175714; doi:10.1016/j.onehlt.2025.101102)
Supplement: Supplementary file 1 — Supplementary material [file mmc1.pdf]

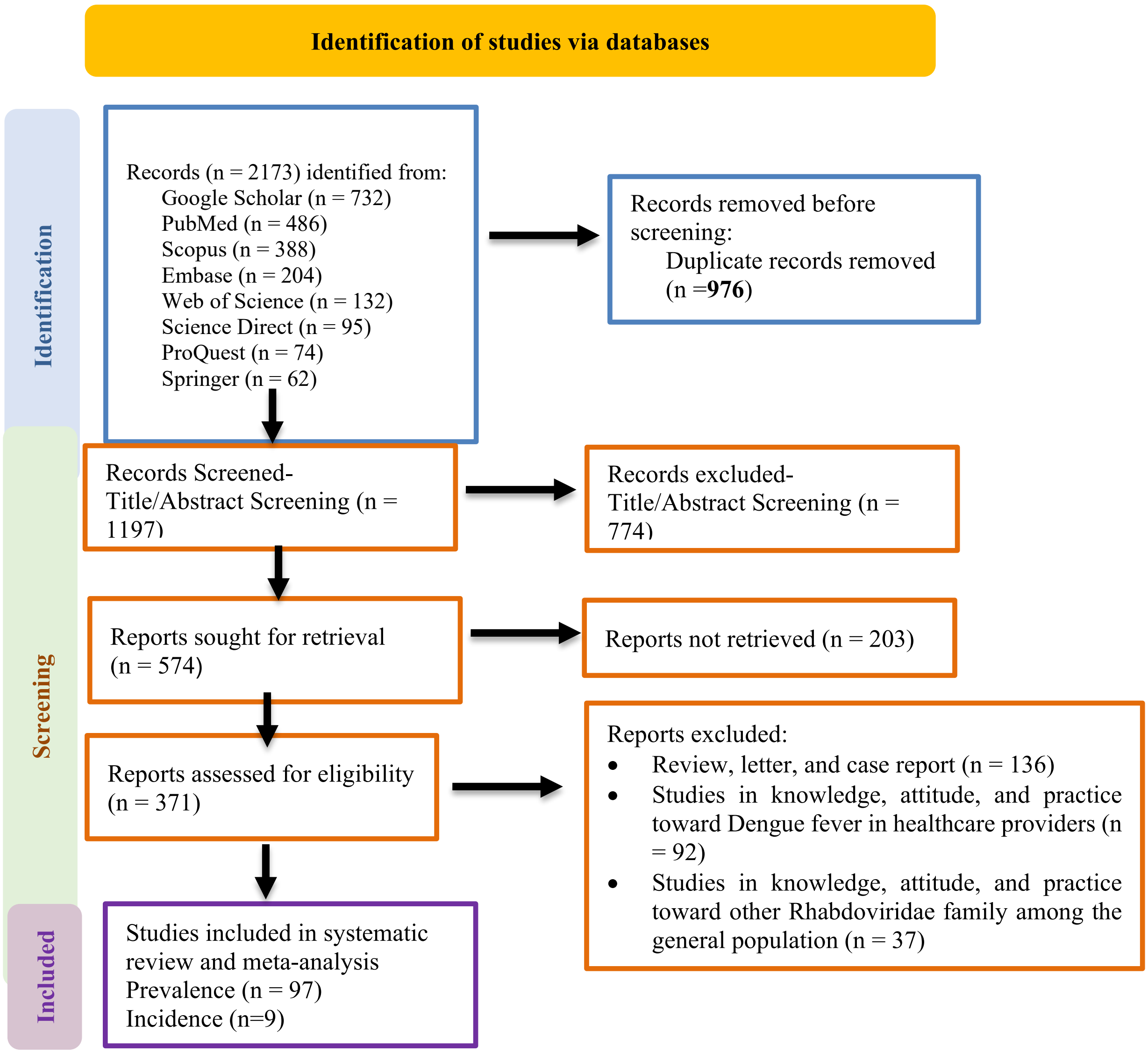

Supplementary Figure 1: PRISMA flowchart of studies included in this systematic review and meta-analysis.

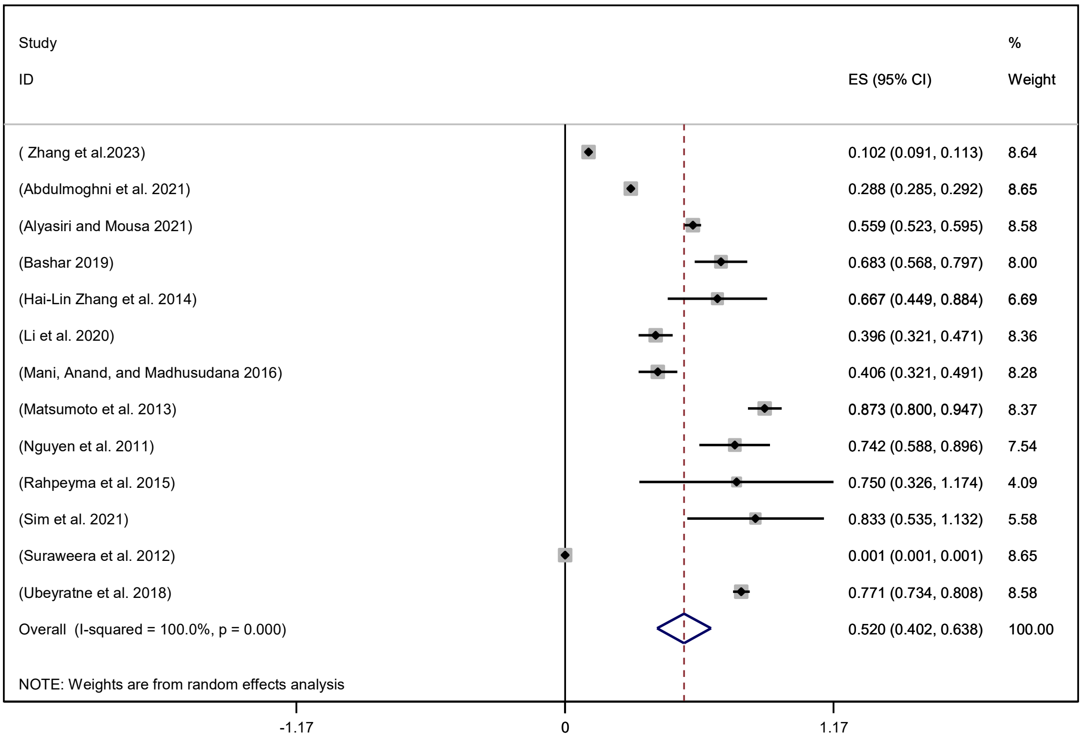

Supplementary Figure 2: Forest plot analysis of rabies diagnostic prevalence in humans in Asian countries.

The x-axis represents the effect size (ES) for rabies prevalence, with values to the right of zero indicating higher prevalence; each square represents a study's estimate, with the square size reflecting the study's weight in the overall analysis.

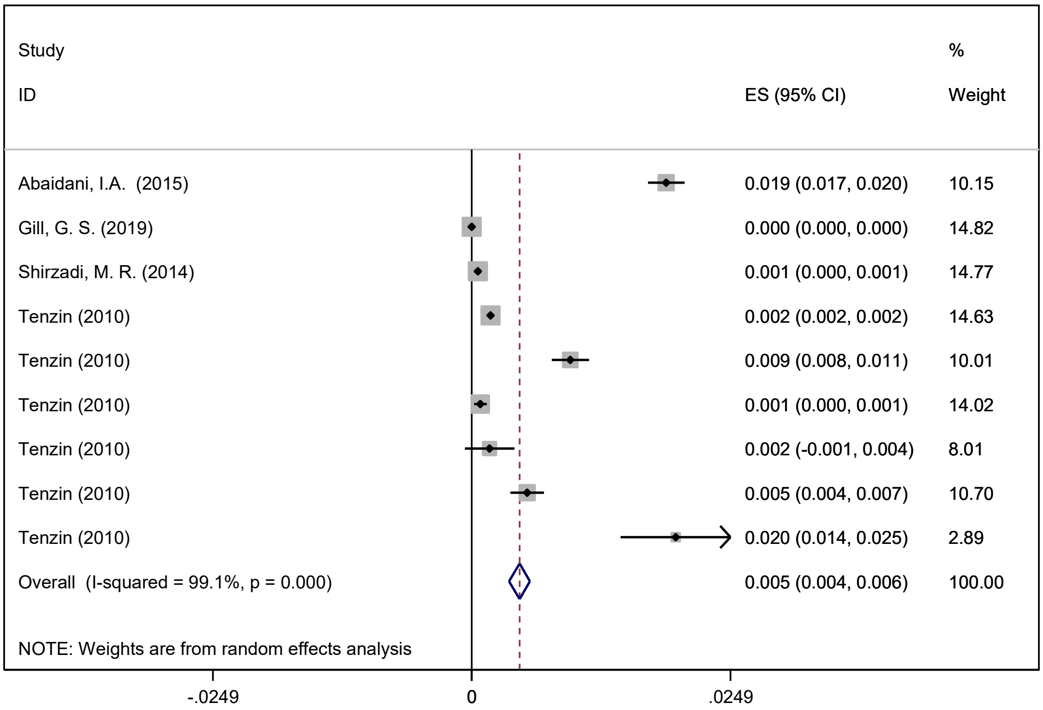

Supplementary Figure 3: Forest plot analysis of rabies incidence among each animal species in Asia. Each study's effect size (ES) and 95% confidence interval (CI) are shown, with square markers indicating individual study estimates; larger squares reflect greater study weight. The diamond at the bottom represents the pooled incidence estimate, with its width denoting the 95% CI. The high I-squared value (99.1%) and significant p-value indicate substantial heterogeneity, suggesting varied rabies incidence rates across the included studies.

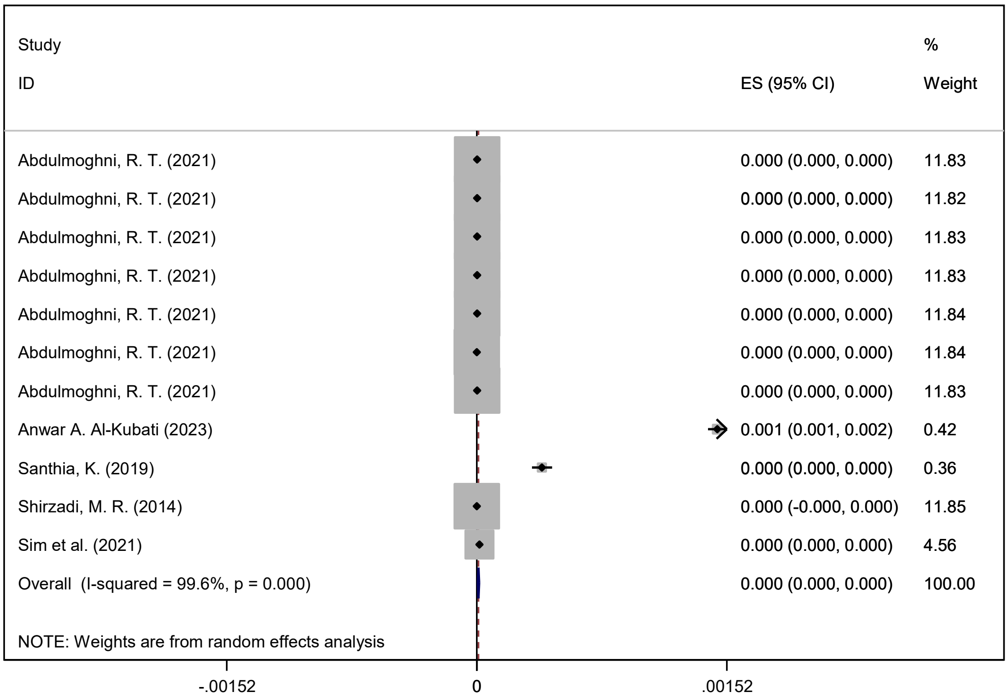

Supplementary Figure 4. Forest plot analysis of rabies incidence among humans based on year of sampling in Asia.

Most studies show an incidence of zero. Only one study reports a minimal non-zero incidence. The pooled estimate, indicated by the diamond, centers around zero, suggesting negligible overall incidence.

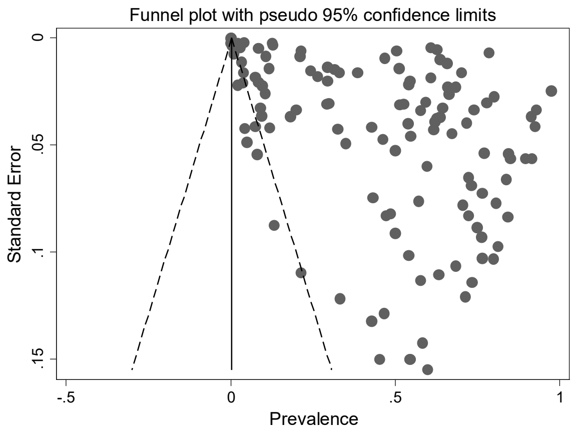

(a)

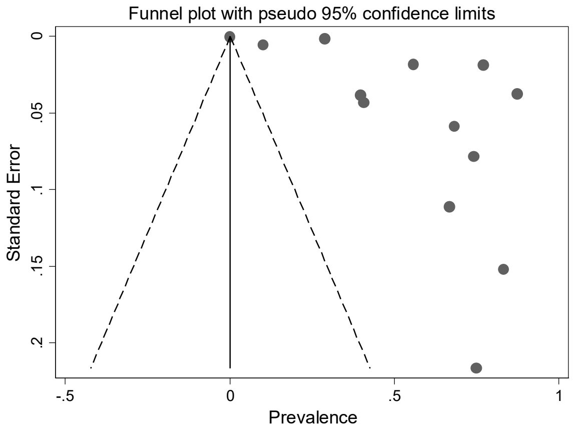

(b)

Supplementary Figure 5: Funnel plots with pseudo 95% confidence interval limits for the detection of publication bias among studies that reported rabies prevalence in animals (a) and humans (b).

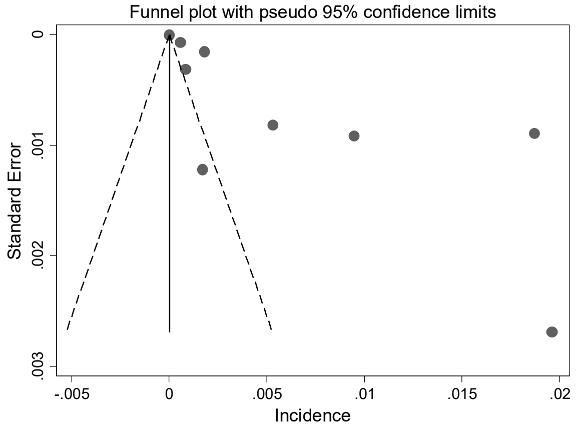

(a)

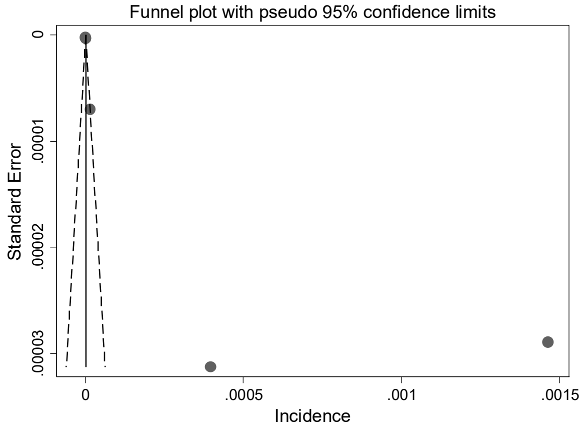

(b)

Supplementary Figure 6: Funnel plots with pseudo 95% confidence interval limits for the detection of publication bias among studies that reported rabies incidence in animals (a) and humans (b).

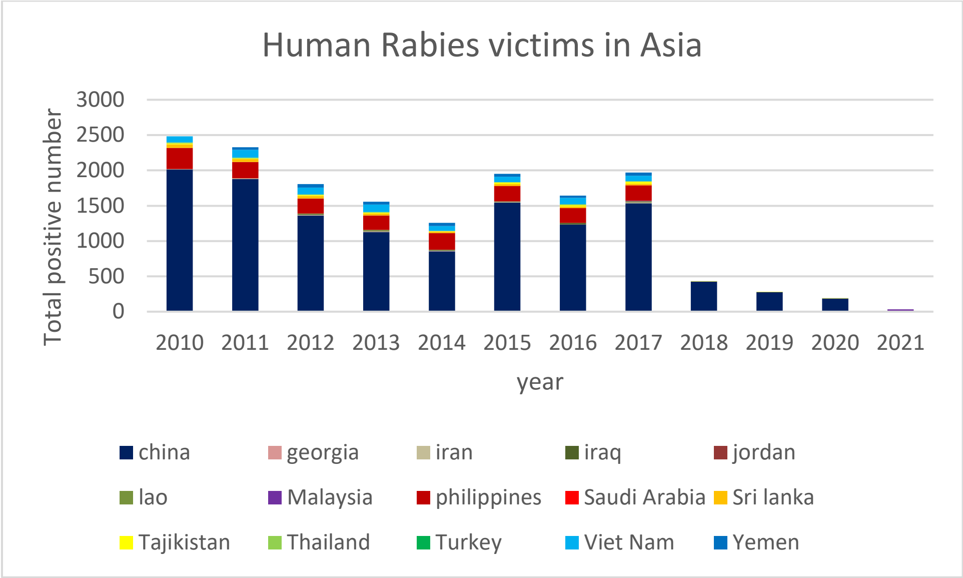

Supplementary Figure 7: WHO Reported of Human Rabies infection during 2010-2021 in Asia.

Supplementary Table 1: Animal studies that have met the eligibility criteria.

Studi ID	Country	Species	Diagnostic method	Total number	Total positive number	QA	Year of sampling
(A. K. T. Nguyen et al. 2011) [1]	Vietnam	Dog	RT-PCR	176	17	6	2007-2009
(A. T. K. Nguyen et al. 2014) [2]	Vietnam	Bat	RT-PCR	789	193	4	2011
(Adelshin et al. 2015) [3]	Mongolia	Animals	RT-PCR	37	18	5	2011-2012
(Afzaal et al. 2013) [4]	Pakistan	Animals	RT-PCR	10	6	4	
(Ahmad et al. 2016) [5]	Pakistan	Dog	Histology	80	3	7	2006-2010
(Ahmed et al. 2015) [6]	Lao PDR	Dog	RT-PCR	1249	640	5	2004-2011
(Al Abaidani et al. 2015) [7]	Oman	Animals/ camel/fox	DFA	61267/61	334/40/47	8	2006-2013
(Al-Eitan et al. 2021) [8]	Jordan	Animals	DFA	12	7	6	2019
(Al-shamahy, Sunhope, and Al-moyed 2013) [9]	Yemen	Dog	DFA	166	104	6	2011
(Bashar 2019) [10]	Iran	Animals	DFA	7448	4663	7	2000-2013
(Beck et al. 2017) [11]	Seri Lanka	Animals	RT-PCR	57	53	7	2007-2011
(Bharathy and Gunaseelan 2016) [12]	India	Dog	Seller's Staining	169	125	5	2011-2013
(Body et al. 2014) [13]	Oman	Animals	DFA	257	135	5	2011-2012
(Brookes et al. 2018) [14]	India	Animals/ buffalo/ cow/dog/ horse	DFA	30/153/110/253/11	15/95/74/130/5	8	2004-2014
(Chang et al. 2015) [15]	Taiwan	Ferret badger	DFA	15	7	5	2010-2013
(D. K. Yang et al. 2012) [16]	Korea S	Animals	DFA	110	20	4	2008-2010
(D. K. Yang et al. 2013) [17]	Korea S	Raccoon dog	FAVN	94	33	5	2011-2012
(D.-K. Yang et al. 2010) [18]	Korea S	dog	NPLA	533	288	6	2006-2007
(Debbarma et al.2023) [19]	India	Animals	DFA	17	13	7	2019-2020
(Douangngeun et al. 2017) [20]	Lao PDR	Dog	DFA	415	284	8	2010-2016
(Ekowati, Sudarnika, and Purnawarman 2020) [21]	Indonesia	Dog	Uk	8641	1116	7	2012-2018
(El-Neweshy et al. 2020) [22]	Oman	Animals	DFA	117	64	6	2017-2019
(Faizee et al. 2012) [23]	Jordan	Animals	DFA	29	21	5	2009-2010
(Feng et al. 2016) [24]	China	Sheep	DFA	140	60	6	2015
(Feng et al. 2020) [25]	China	Animals/ dog	DFA	185/10118	144/33	9	2004-2018
(Feng et al. 2021) [26]	China	Animals	DFA	212	170	4	2010-2020
(Feng et al.2022) [27]	Mongolia	Animals/ fox/ cow/sheep	DFA	16/19/40/15	13/16/39/11	8	2019-2021
(Gill et al. 2019) [28]	India	animals	DFA	41	30	5	2016-2017
(H. Liu et al. 2020) [29]	China	Animals	RT-PCR	1222	34	4	2017-2019

(Hai-Lin Zhang et al. 2014) [30]	China	Dog	DFA	1295	139	6	2008-2010
(Hananeh et al. 2015) [31]	Jordan	Animals	DFA	11	6	6	2012-2013
(Horton et al. 2013) [32]	Iraq	Animals	DFA	40	3	8	2010-2011
(Hosseini Heydarabadi et al. 2020) [33]	Iran	Animals	DFA	1637	1078	6	2015-2017
(Ismail et al. 2020) [34]	Iraq	Animals	DFA	58	53	6	2013-2017
(J.L. Cruz et al.2023) [35]	Philippines	Animals/ Dog/ Cat	DFA	19/682/77	12/415/7	8	2021-2022
(Jayasundara 2020) [36]	Seri Lanka	Cat	DFA	2335	490	4	2010-2015
(Jiang et al. 2010) [37]	China	Bat	DFA	183	2	7	2005-2006
(Jiao et al. 2013) [38]	China	Dog/Cat/cow/mouse	Uk	3726/20/14/45	83/1/3/1	6	2005-2011
(Kartskhia et al. 2020) [39]	Georgia	Animals	DFA	4331	932	4	2009-2018
(Kasem et al. 2019) [40]	Saudi Arabia	Camel/cat/ dog/fox/ goat/sheep	DFA	40/19/47/24/ 29/31	34/13/34/18/26/26	7	2010-2017
(Kavoosian et al. 2023) [41]	Iran	Animals/ cow/ dog	DFA	26/128/34	21/92/26	8	2016-2018
(Kim et al. 2011) [42]	Korea S	Dog	Uk	500	147	7	2010
(Kimitsuki et al. 2020) [43]	Philippines	Animals	DFA	156	84	6	2019
(Kumarasinghe KADM et al. 2017 2017) [44]	Seri Lanka	Dog/cat	DFA	426/216	281/63	3	2015-2016
(Lachica, Murao, and Mata 2018) [45]	Philippines	Dog	DFA	598	158	8	2006-2017
(Lapiz et al. 2012) [46]	Philippines	Dog	DFA	148	6	7	2007-2010
(Lu et al. 2013) [47]	China	Bat	RT-PCR	2969	85	8	2003-2008
(Mananggit et al. 2021) [48]	Philippines	Cat/Dog	DFA	25/266	2/158	8	2019-2020
(Mani et al. 2017) [49]	India	Bat	DFA	164	0	5	2013-2014
(Manjunatha Reddy et al. 2018) [50]	India	Animals	DFA	140	28	4	2013-2016
(Matsumoto et al. 2017) [51]	Seri Lanka	Animals	RT-PCR	44	19	7	2009-2012
(Memish, Assiri, and Gautret 2015) [52]	Saudi Arabia	Animals	Histological	40	37	4	2005-2010
(MIAO et al. 2018) [53]	China	Ferret badger	DFA	1407	43	4	2014-2016
(Mondal and Yamage 2014) [54]	Bangladesh	Buffalo/ cow/goat/ sheep	Uk	139737/2900621/1545831 /141707	129/9056/4502/398	6	2010-2012
(Montemayor and Quipanes 2015) [55]	Philippines	Dog	Uk	24	13	4	2009-2013
(Muminov et al. 2021) [56]	Tajikistan	Animals	RT-PCR	59	7	4	2016-2018
(Nadalian MGh, Tadjbakhsh H, Mokhber Dezfuli MR, Rezakhani A, Simani S 2016) [57]	Iran	Animals	uk	2230	1421	3	2001-2004
(Namwongprom et al. 2020) [58]	Thailand	Animals	DFA	8375	352	4	2013-2014
(NinidzeL. and MenteshashviliS 2013) [59]	Georgia	Animals	Uk	36	17	3	2006-2012

(P et al. 2016) [60]	Thailand	Animals	DFA	120	39	6	2011-2014
(Pal et al. 2021) [61]	Nepal	Animals	DFA	2771	1302	8	2005-2017
(Pant 2014) [62]	Nepal	Animal/ buffalo/ cow/dog/ goat	DFA	168/11/14/128/15	107/6/10/79/12	4	2008-2012
(Prabhu et al. 2018) [63]	India	Cow/dog	DFA	21/211	16/136	5	2012-2014
(Rahpeyma et al. 2015) [64]	Iran	Cow/ animals/ dog/sheep	DFA	45/42/90/14	38/24/45/6	4	2015
(Ranasinghe 2017) [65]	Seri Lanka	Animals	DFA	6860	3455	9	2011-2015
(Shao et al. 2011) [66]	China	Raccoon dog	DFA	15	5	6	2007
(Sharma, Singh, and Narang 2015) [67]	India	Animals	DFA	34	24	6	2015
(Singh, Islam, and Hasan 2015) [68]	Bangladesh	Dog	Uk	511	60	7	2013-2014
(Th et al. 2017) [69]	Taiwan	Ferret badger	DFA	788	553	7	2013-2016
(Thanapongtharm et al. 2021) [70]	Thailand	Animals	DFA	18175	2323	8	2017-2018
(Tsai et al. 2016) [71]	Taiwan	Animals	DFA	831	276	5	2013
(Tuvshintulga et al. 2015) [72]	Mongolia	Animals	DFA	24	22	6	2008-2010
(Ubeyratne et al. 2018) [73]	Seri Lanka	Dog	Uk	11288	6885	6	2004-2016
(Vos et al. 2014) [74]	Turkey	Cow	DFA	881	340	5	2008-2011
(Vu et al. 2021) [75]	Vietnam	Dog	DFA	876	7	7	2015-2017
(Wang et al. 2013) [76]	China	Ferret badger/ dog	DFA	64/201	6/15	6	2008-2009
(Wera et al. 2013) [77]	Indonesia	Dog	DFA	3346	2628	4	2000-2011
(Wilde et al. 2012) [78]	Thailand	Animal/cat/ cow/dog	Uk	1138/141/19/978	334/15/11/308	4	2009
(Y. Liu et al. 2010) [79]	China	Dog/ferret badger	DFA	15/265	2/9	8	2010
(Y. Liu et al. 2013) [80]	China	Bat	DFA	261	1	7	2012
(Yale et al. 2019) [81]	India	Animals	DFA	209	121	5	2018
(Yu et al. 2010) [82]	China	Dog	DFA	3007	254	5	
(Zeynalova et al. 2015) [83]	Azerbaijan	Animals	RT-PCR	326	216	8	2000-2010
(Zhang et al. 2010) [84]	China	Ferret Badger	DFA	23	1	7	2009
(Zhang et al. 2013) [85]	China	Ferret Badger	DFA	178	15	4	2007-2009
(ZHANG et al. 2017) [86]	China	Dog	DFA	224	67	7	2015
(Zhu et al. 2011) [87]	China	Sheep	RT-PCR	110	51	7	2010

DFA: direct fluorescence anti

Body test; RT-PCR: Reverse transcription polymerase chain reaction

UK: unknown,

NPLA: neutralizing peroxidase-linked antibody

FAVN: Fluorescent Antibody Virus Neutralization

QA: Quality assessment

Supplementary Table 2: Human studies have met the eligibility criteria of this analysis

Study ID	Country	Diagnostic method	Total number	Total positive number	QA	Sampling year
(Zhang et al.2023) [88]	China	DFA	3032	310	7	2015-2021
(Abdulmoghni et al. 2021) [89]	Yemen	uk	76049	21927	7	2011-2017
(Alyasiri and Mousa 2021) [90]	Iraq	DFA	732	409	4	2019
(Bashar 2019) [10]	Iran	DFA	63	43	7	2000-2013
(Hai-Lin Zhang et al. 2014) [91]	China	DFA	18	12	6	2008-2010
(Li et al. 2020) [92]	China	RT-PCR	164	65	7	2005-2019
(Mani, Anand, and Madhusudana 2016) [93]	India	RT-PCR	128	52	9	2012-2014
(Matsumoto et al. 2013) [94]	Seri Lanka	DFA	79	69	8	2008-2010
(Nguyen et al. 2011) [95]	Vietnam	RT-PCR	31	23	6	2007-2009
(Rahpeyma et al. 2015) [64]	Iran	DFA	4	3	4	2015
(Sim et al. 2021) [96]	Malaysia	RT-PCR	6	5	9	2020-2021
(Suraweera et al. 2012) [97]	India	uk	122429	95	8	2001-2003
(Ubeyratne et al. 2018) [73]	Seri Lanka	MRI	502	387	6	2003-2016

DFA: direct fluorescence anti

Body test; RT-PCR: Reverse transcription polymerase chain reaction

UK: unknown,

MRI: Magnetic resonance imaging

QA: Quality assessment

Supplementary Table 3: Animal studies incidence

Studi ID	Country	Species	Diagnostic Method	Total Number	Total Positive Number	QA	year of sampling
(Shirzadi, M. R 2014). [98]	Iran	Dog	RT-PCR	105896	62	6	2009-2010
(Al Abaidani et al. 2015) [7]	Oman	Dog	RT-PCR	22722	425	6	1991-2014
(Gill et al. 2019) [28]	India	Dog	DFA	8100000	30	6	2016-2017
(Tenzin 2010) [99]	Bhutan	Cow/ dog/ horse/ cat	UK	77832/11161/8357/1156	141/106/7/2	6	2005-2007
(Tenzin 2010) [100]	Bhutan	Cow/ dog	UK	7864/2651	42/52	6	2008

DFA: direct fluorescence anti

Body test; RT-PCR: Reverse transcription polymerase chain reaction

UK: unknown,

QA: Quality assessment

Supplementary Table 4: Human studies incidence

Study ID	Country	Diagnostic method	Total number	Total positive number	QA	Sampling Year
(Shirzadi, M. R. 2014) [98]	Iran	RT-PCR	8466633	3	7	2009-2010
(Abdulmoghni et al. 2021) [89]	Yemen	RT-PCR	152759721	295	7	2011-2017
(Santhia, K 2019) [101].	Indonesia	RT-PCR	405627	161	7	2013-2018
(Sim et al. 2021) [102]	Malaysia	RT-PCR	350000	6	6	2020-2021
(Anwar A. Al-Kubati 2023) [103]	Yemen	RT-PCR	1745549	2555	8	2011-2017

Body test; RT-PCR: Reverse transcription polymerase chain reaction
QA: Quality assessment

References:

[1] A.K.T. Nguyen, D. V Nguyen, G.C. Ngo, T.T. Nguyen, S. Inoue, A. Yamada, X.K. Dinh, D. V Nguyen, T.X. Phan, B.Q. Pham, Molecular epidemiology of rabies virus in Vietnam (2006–2009), Jap. J. of Infect. Dis. 64 (2011) 391–396.

[2] A.T.K. Nguyen, T.T. Nguyen, A. Noguchi, D.V. Nguyen, G.C. Ngo, V.D. Thong, B. Olowokure, S. Inoue, Bat lyssaviruses, northern Vietnam, Emerg. Infect. Dis. 20 (2014) 161. https://doi.org/10.3201/eid2001.130813

[3] R. V Adelshin, O. V Melnikova, Y.N. Trushina, A.D. Botvinkin, T.I. Borisova, E.I. Andaev, D.B. Verzhutsky, A.S. Khangazhinov, S. V Balakhonov, A new outbreak of fox rabies at the Russian–Mongolian border, Virologica Sinica 30 (2015) 313–315. https://doi.org/10.1007/s12250-015-3609-0

[4] S.A. Shahida Afzaal, M.N. Muhammad Numan, Z.-A.Q. Zafar-ul-Ahsan Qureshi, M.S. Munazza Shaukat, I.A. Khan, Z.H. Zaheer Hussain, Z.-A. Zain-ul-Abidin, A.W. Manzoor, M.H. Mudasser Habib, Ante-mortem diagnosis of rabies in cows and buffaloes, J of Adv Vet Res 3 (2013) 27-30. Retrieved from https://advetresearch.com/index.php/AVR/article/view/110

[5] W. Ahmad, F. Mahmood, Y. Li, M. Duan, Z. Guan, M. Zhang, M.A. Ali, Z. Liu, Immunopathological studies of canine rabies in Faisalabad, Pakistan, J. of Anim. and Plant Sci 26 (2016) 636-642.

[6] K. Ahmed, P. Phommachanh, P. Vorachith, T. Matsumoto, P. Lamaningao, D. Mori, M. Takaki, B. Douangngeun, B. Khambounheuang, A. Nishizono, Molecular Epidemiology of Rabies Viruses Circulating in Two Rabies Endemic Provinces of Laos, 2011–2012: Regional Diversity in Southeast Asia, PLoS Negl Trop Dis 9 (2015). https://doi.org/10.1371/journal.pntd.0003645.

[7] I. Al Abaidani, S. Al Abri, K.P. Prakash, M.H. Hussain, M.H. Hussain, A.H. Al Rawahi, Epidemiology of rabies in Oman: a retrospective study (1991–2013), East. Medit. Health J. 21 (2015) 591–597. https://doi.org/10.26719/2015.21.8.591.

[8] L.N. Al-Eitan, G. Wu, M. Golding, Y. Tang, H. Goharriz, D.A. Marston, A.R. Fooks, L.M. McElhinney, Whole-genome sequencing and phylogenetic analysis of rabies viruses from Jordan, PLoS Negl. Trop. Dis. 15 (2021) e0009431. https://doi.org/10.1371/journal.pntd.0009431.

[9] H.A. Al-Shamahy, A. Sunhope, K.A. Al-Moyed, Prevalence of rabies in various species in Yemen and risk factors contributing to the spread of the disease, Sultan Qaboos Univers. Med. J. 13 (2013) 404. https://doi.org/10.12816/0003263.

[10] R. Bashar, Spatial Epidemiology of Rabies in Iran, (2019). http://nbn-resolving.de/urn:nbn:de:kobv:188-refubium-24489-8. Accessed 2025/05/30.

[11] S. Beck, P. Gunawardena, D.L. Horton, D.J. Hicks, D.A. Marston, A. Ortiz-Pelaez, A.R. Fooks, A. Núñez, Pathobiological investigation of naturally infected canine rabies cases from Sri Lanka, BMC Vet. Res. 13 (2017) 1–9. https://doi.org/10.1186/s12917-017-1024-5.

[12] S. Bharathy, L. Gunaseelan, Study on epidemiological trends of canine rabies between 2011 and 2014 in Chennai city, South India, Ind. J. of Anim. Res. 50 (2016) 964–968. https://doi.org/10.18805/ijar.11424

[13] M.H.H. Body, A. Al Rawahi, M.H. Hussain, S.S. Al Habsi, A.A. Wadir, N. Saravanan, M.S. Ahmed, S. Rajamony, Study on molecular characterization of rabies virus N gene segment from different animal species in the Sultanate of Oman, J. of Vet. Med. and Anim. Health 6 (2014) 295–301. https://doi.org/10.5897/jvmah2014.0326.

[14] V.J. Brookes, G.S. Gill, C.K. Singh, B.S. Sandhu, N.K. Dhand, B.B. Singh, J.P.S. Gill, M.P. Ward, Exploring animal rabies endemicity to inform control programmes in

Punjab, India, Zoonoses and Public Health 65 (2018) e54–e65. https://doi.org/10.1111/zph.12409.
[15] J.C. Chang, K.J. Tsai, W.C. Hsu, Y.C. Tu, W.C. Chuang, C.Y. Chang, S.W. Chang, T.E. Lin, K.Y. Fang, Y.F. Chang, H.J. Tsai, S.H. Lee, Rabies virus infection in ferret badgers (Melogale moschata subaurantiaca) in Taiwan: A retrospective study, J. of Wildl. Dis. 51 (2015) 923–928. https://doi.org/10.7589/2015-04-090.
[16] D.K. Yang, E.K. Shin, Y.I. Oh, K.W. Lee, C.S. Lee, S.Y. Kim, J.A. Lee, J.Y. Song, Comparison of four diagnostic methods for detecting rabies viruses circulating in Korea, J. of Vet. Sci. 13 (2012) 43–48. https://doi.org/10.4142/jvs.2012.13.1.43.
[17] D.-K. Yang, H.-H. Kim, J.-J. Nah, S.-S. Choi, J.-T. Kim, W.-H. Jeong, J.-Y. Song, Serologic survey of rabies virus, canine distemper virus and parvovirus in wild raccoon dogs (Nyctereutes procyonoides koreensis) in Korea, J. of Bact. and Virol. 43 (2013) 204–209. http://dx.doi.org/10.4167/jbv.2013.43.3.204.
[18] D.K. Yang, S.S. Yoon, K.K. Lee, J.W. Byun, Y.C. Bae, Y.I. Oh, J.Y. Song, Rabies immune status in the stray and companion dogs in Korea, Kor. J. of Vet. Res. (50 (2010) 133–137.
[19] B. Debbarma, J.B. Dutta, S. Isloor, B.C.H. Baishya, P.K.R. Boro, T. Das, Incidence of Rabies in animals of Kamrup Metro district of Assam, India, The Pharma Innov. J. 12 (2023) 169–172.
[20] B. Douangngeun, W. Theppangna, P. Phommachanh, K. Chomdara, S. Phiphakhavong, S. Khounsy, M. Mukaka, D.A.B. Dance, S.D. Blacksell, Rabies surveillance in dogs in Lao PDR from 2010-2016, PLoS Negl. Trop. Dis. 11 (2017) 1–12. https://doi.org/10.1371/journal.pntd.0005609.
[21] R.V. Ekowati, E. Sudarnika, T. Purnawarman, Spatial analysis of rabies cases in dogs in Bali Province, Indonesia, Adv. in Anim. and Vet. Sci. 8 (2020) 32–40. https://doi.org/10.17582/journal.aavs/2020/8.1.32.40.
[22] M.S. El-Neweshy, N. Al Mayahi, W. Al Mamari, Z. Al Rashdi, J.H. Al Mawly, Animal rabies situation in Sultanate of Oman (2017–2019), Trop. Anim. Health and Product. 52 (2020) 3069–3076. https://doi.org/10.1007/s11250-020-02328-0.
[23] N. Faizee, N.Q. Hailat, M.M.K. Ababneh, W.M. Hananeh, A. Muhaidat, Pathological, Immunological and Molecular Diagnosis of Rabies in Clinically Suspected Animals of Different Species Using Four Detection Techniques in Jordan, Transb. Emerg. Dis. 59 (2012) 154–164. https://doi.org/10.1111/j.1865-1682.2011.01255.x.
[24] Y. Feng, Y. Shi, M. Yu, W. Xu, W. Gong, Z. Tu, L. Ding, B. He, H. Guo, C. Tu, Livestock rabies outbreaks in Shanxi province, China, Arch. of Virol. 161 (2016) 2851–2854. https://doi.org/10.1007/s00705-016-2982-9.
[25] Y. Feng, Y. Wang, W. Xu, Z. Tu, T. Liu, M. Huo, Y. Liu, W. Gong, Z. Zeng, W. Wang, Animal rabies surveillance, China, 2004–2018, Emerg. Infect. Dis. 26 (2020) 2825. https://doi.org/10.3201/eid2612.200303.
[26] Y. Feng, J. Ma, S. Sun, L. Chi, Z. Kou, C. Tu, Epidemiology of Animal Rabies — China, 2010–2020, China CDC Wkly 3 (2021) 815–818. https://doi.org/10.46234/ccdcw2021.202.
[27] Y. Feng, Y. Wang, Hada, Deijide, Gaosuyilatu, X. Li, Z. Xu, Hasibagen, A. Bulage, L. Li, Diversity of rabies virus detected in Inner Mongolia, China, 2019–2021, Transbound. Emerg. Dis. 69 (2022) 249–253. https://doi.org/10.1111/tbed.14451.
[28] G.S. Gill, B.B. Singh, N.K. Dhand, R.S. Aulakh, B.S. Sandhu, M.P. Ward, V.J. Brookes, Estimation of the incidence of animal rabies in Punjab, India, PloS One 14 (2019) e0222198. https://doi.org/10.1371/journal.pone.0222198.

[29] H. Liu, L. Li, X. Yuan, X. Si, M. Zhang, M. Duan, N. Shi, Rabies viruses in specific wild fur animals in northern China, 2017–2019, Transbound. Emerg. Dis. 67 (2020) 2307–2312. https://doi.org/10.1111/tbed.13629.
[30] H.-L. Zhang, Y.-Z. Zhang, W.-H. Yang, X.-Y. Tao, H. Li, J.-C. Ding, Y. Feng, D.-J. Yang, J. Zhang, J. He, Molecular epidemiology of reemergent rabies in Yunnan Province, southwestern China, Emerg. Infect. Dis. 20 (2014) 1433. https://doi.org/10.3201/eid2009.130440.
[31] W.M. Hananeh, I.M. Nassir, M.M.K. Ababneh, N.Q. Hailat, C.C. Brown, Pathological and molecular diagnosis of rabies in clinically suspected food animals using different diagnostic tests, Large Anim. Rev. 21 (2015) 243–250.
[32] D.L. Horton, M.Z. Ismail, E.S. Siryan, A.R.A. Wali, H.E. Ab-dulla, E. Wise, K. Voller, G. Harkess, D.A. Marston, L.M. McElhinney, S.F. Abbas, A.R. Fooks, Rabies in Iraq: Trends in Human Cases 2001-2010 and Characterisation of Animal Rabies Strains from Baghdad, PLoS Negl. Trop. Dis. 7 (2013). https://doi.org/10.1371/journal.pntd.0002075.
[33] F. Hosseini Heydarabadi, K. Baessi, R. Bashar, M. Fazeli, F. Sheikholeslami, A phylogenetic study of new rabies virus strains in different regions of Iran, Virus Genes 56 (2020) 361–368. https://doi.org/10.1007/s11262-020-01752-6.
[34] M.Z. Ismail, N.K. Al-Hamdi, A.N. Al-Amery, D.A. Marston, L. McElhinney, E. Taylor, V. del Rio Vilas, T.M. Dadan, A.R. Fooks, D.L. Horton, Quantifying and mapping the burden of human and animal rabies in Iraq, PLoS Negl. Trop. Dis. 14 (2020) e0008622. https://doi.org/10.1371/journal.pntd.0008622,
[35] J.L. Cruz, A.M. Garcia, N. Saito, M.G.O. Lagayan, R.C. Dela Pena, M.S. Usana, S.P. Agustin, J.Z. Tattao, C. V Mamauag, O.P. Ducayag, Evaluation of lateral flow devices for postmortem rabies diagnosis in animals in the Philippines: a multicenter study, J. Clin, Microbiol. 61 (2023) e00842-23. https://doi.org/10.1128/jcm.00842-23.
[36] V.K. Jayasundara, Public awareness on vaccination of cats against rabies and risk of human rabies due to cats in Sri Lanka, Int. J. Infect. Dis. 101 (2020) 249.
[37] Y. Jiang, L. Wang, Z. Lu, H. Xuan, X. Han, X. Xia, F. Zhao, C. Tu, Seroprevalence of rabies virus antibodies in bats from Southern China, Vector Borne Zoonotic Dis.10 (2010) 177–181. https://doi.org/10.1089/vbz.2008.0212.
[38] W. Jiao, H. Li, X. Tao, M. Song, X. Shen, Z. Guo, Y. Zhao, Q. Tang, G. Liang, Investigation and analysis of rabies viral infection and distribution in China in 2005-2012, Virol. Sin. 28 (2013) 183–185. https://doi.org/10.1007/s12250-013-3324-7.
[39] N. Kartskhia, L. Ninidze, T. Chaligava, I. Menteshashvili, Rabies public surveillance program in Georgia, Int. J. Infect. Dis. 101 (2020) 543.
[40] S. Kasem, R. Hussein, A. Al-Doweriej, I. Qasim, A. Abu-Obeida, I. Almulhim, H. Alfarhan, A.A. Hodhod, M. Abel-latif, O. Hashim, D. Al-Mujalli, A. AL-Sahaf, Rabies among animals in Saudi Arabia, J. Infect. Public Health 12 (2019) 445–447. https://doi.org/10.1016/j.jiph.2018.10.005.
[41] S. Kavoosian, R. Behzadi, M. Asouri, A.A. Ahmadi, M. Nasirikenari, A. Salehi, O.F. Bayramlar, Comparison of rabies cases received by the sho mal Pasteur Institute in northern Iran: a 2-year study, Glob. Health Epidemiol. and Genom. 2023 (2023) e10. https://doi.org/10.1155/2023/3492601.
[42] N.-H. Kim, H.-S. Chae, H.-R. Son, Y.-I. Kang, J.-H. Lee, S.-G. Kim, Serological survey of rabies virus from the stray dogs in Seoul, Kor. J. Vet. Serv. 34 (2011) 297–301. https://doi.org/10.7853/kjvs.2011.34.4.297.
[43] K. Kimitsuki, N. Saito, K. Yamada, C.H. Park, S. Inoue, M. Suzuki, M. Saito-Obata, Y. Kamiya, D.L. Manalo, C.S. Demetria, M.R. Mananggit, B.P. Quiambao, A. Nishizono, Evaluation of the diagnostic accuracy of lateral flow devices as a tool to

diagnose rabies in post-mortem animals, PLoS Negl. Trop. Dis. 14 (2020) e0008844. https://doi.org/10.1371/journal.pntd.0008844.
[44] K.A.D.M Kumarasinghe, S. Nanayakkara , R. Balasubramaniam , A.U. Jayasinghe, G.K.J.N Udara, K.A.D.N. Perera, Prospective study to ascertain the relationship between rabies infection and anti-rabies immunization status of dogs and cats in Sri Lanka, oral presentation at the 26th Annual Scientific Sessions of the Sri Lanka College of Microbiologists, 31 August – 1 September 2017.
[45] Z.P.T. Lachica, S.A. Evangelio, E.O.Diamante, A.J. Clemente, J.M. Peralta, L.A.E. Murao, M.A.E. Mata, P.A. Alviola, Trends of Canine Rabies Lyssavirus and Impact of the Intensified Rabies Control Program in Davao City , Philippines : 2006 – 2017, Philippine J. Sci. 148 (2018) 751-763.
[46] S.M.D. Lapiz, M.E.G. Miranda, R.G. Garcia, L.I. Daguro, M.D. Paman, F.P. Madrinan, P.A. Rances, D.J. Briggs, Implementation of an intersectoral program to eliminate human and canine rabies: the Bohol Rabies Prevention and Elimination Project, PLoS Neglect. Trop. Dis. 6 (2012) e1891. https://doi.org/10.1371/journal.pntd.0001891.
[47] Z.L. Lu, W. Wang, W.L. Yin, H.B. Tang, Y. Pan, X. Liang, Q. Liu, Y. Xiong, N. Minamoto, T.R. Luo, Lyssavirus surveillance in bats of southern China’s Guangxi Province, Virus Genes 46 (2013) 293–301. https://doi.org/10.1007/s11262-012-0854-2.
[48] M.R. Mananggit, K. Kimitsuki, N. Saito, A.M.G. Garcia, P.M.T. Lacanilao, J.T. Ongtangco, C.R. Velasco, M.V.D. Rosario, M.G.O. Lagayan, K. Yamada, Background and descriptive features of rabies-suspected animals in Central Luzon, Philippines, Trop. Med. Health 49 (2021) 1–9. https://doi.org/10.1186/s41182-021-00351-x.
[49] R.S. Mani, D.P. Dovih, M.A. Ashwini, B. Chattopadhyay, P.K. Harsha, K.M. Garg, S. Sudarshan, R. Puttaswamaiah, U. Ramakrishnan, S.N. Madhusudana, Serological Evidence of Lyssavirus Infection among Bats in Nagaland, a North-Eastern State in India, Epidemiol. Infect. 145 (2017) 1635–1641. https://doi.org/10.1017/S0950268817000310.
[50] G.B. Manjunatha Reddy, S. Krishnappa, B. Vinayagamurthy, R. Singh, K.P. Singh, M. Saminathan, B. Sajjanar, H. Rahman, Molecular epidemiology of rabies virus circulating in domestic animals in India, Virusdisease 29 (2018) 362–368. https://doi.org/10.1007/s13337-018-0478-9.
[51] T. Matsumoto, S. Nanayakkara, D. Perera, S. Ushijima, O. Wimalaratne, A. Nishizono, K. Ahmed, Terrestrial animal-derived rabies virus in a juvenile Indian flying fox in Sri lanka, Jpn. J. Infect. Dis. 70 (2017) 693–695. https://doi.org/10.7883/yoken.JJID.2017.249.
[52] Z.A. Memish, A.M. Assiri, P. Gautret, Rabies in Saudi Arabia: a need for epidemiological data, Int. J. Infect. Dis. 34 (2015) 99–101. https://doi.org/10.1016/j.ijid.2015.03.016.
[53] M.F. Miao FaMing, C.T. Chen Teng, L.Y. Liu Ye, Z.S. Zhang ShouFeng, Z.F. Zhang Fei, L.N. Li Nan, H.R. Liang, Emerging new phylogenetic groups of rabies virus in Chinese ferret badgers, Biomed. Environ. Sci. 31 (2018) 479-482. https://doi.org/10.3967/bes2018.064.
[54] S.P. Mondal, M. Yamage, A retrospective study on the epidemiology of anthrax, foot and mouth disease, haemorrhagic septicaemia, peste des petits ruminants and rabies in Bangladesh, 2010-2012, PLoS ONE 9 (2014), e104435. https://doi.org/10.1371/journal.pone.0104435.

[55] M.L.B. Montemayor, A.C. Quipanes, Animal Rabies in Bukidnon, Philippines: Status and Assessment of Its Control, 2015, the 4th food safety and zoonoses symposium for Asia Pacific, 3-5 August 2015.
[56] A.A. Muminov, G.N. Mamadotokhonova, F.B. Kamolzoda, O.G. Petrova, V.M. Usevich, I.M. Milshtein, Issues and Epidemiological Aspects of the Rabies Disease in South-Eastern Tajikistan, International Transaction Journal of Engineering, Management, & Applied Sciences & Technologies 12 (2021) 1–9. http://doi.org/10.14456/ITJEMAST.2021.121.
[57] M.Gh. Nadalian, H. Tadjbakhsh, M.R. Mokhber-Dezfuli, A. Rezakhani, S. Simani, M. Bolourchi, Rabies and its present situation in Iran, Iranian J. Rumin. Health Res. 1 (2016) 32-34. https://doi.org/10.22055/ijrhr.2017.12845.
[58] K. Namwongprom, K. Angchokchatchawan, T. Niomoh, A. Wiratsudakul, The Nationwide Surveillance of Animal Rabies in Thailand in 2013-2014 and Its Implications on Human Rabies Prevention and Control, News.Ge (2020) https://news.ge/anakliis-porti-aris-qveynis-momava.
[59] L. Ninidze, S. Menteshashvili, Rabies in Georgia, Ветеринарна Медицина (2013) 51.
[60] B. Puyati, S. Senayai, K. Chanachai, P. Panichabhongse, Epidemiological and genetic characteristics of rabies virus in Ubon Ratchathani province, Thailand, 2011-2014, Outbreak, Surveillance and Investigation Reports 9 (2016) 8–14.
[61] P. Pal, A. Yawongsa, R. Bhatta, H. Shimoda, T. Rukkwamsuk, Animal rabies epidemiology in Nepal from 2005 to 2017, Int. J. One Health 7 (2021) 6.pdf.
[62] G.R. Pant, Molecular epidemiology of rabies virus in Nepal, Intern. J. Infect. Dis. 21 (2014) 195. https://doi.org/10.1016/j.ijid.2014.03.828.
[63] K.N. Prabhu, S. Isloor, B.H. Veeresh, D. Rathnamma, R. Sharada, L.J. Das, M.L. Satyanarayana, N.R. Hegde, S.A. Rahman, Application and comparative evaluation of fluorescent antibody, immunohistochemistry and reverse transcription polymerase chain reaction tests for the detection of rabies virus antigen or nucleic acid in brain samples of animals suspected of rabies in India, Vet. Sci. 5 (2018) 24. https://doi.org/10.3390/vetsci5010024.
[64] M. Rahpeyma, F. Fahartaj, M. Fazeli, F. Sheykholeslami, R. Bashar, N. Howeizeh, A. Gholami, Epidemiological study of rabies infection in specimens sent to pasteur institute of Iran in 2015, J. Babol Univ. Med. Sci 17 (2015) 65–70. http://dx.doi.org/10.22088/jbums.17.12.65.
[65] S. Ranasinghe, The rabies epidemic in Sri Lanka (2017) Thesis University of Veterinary Medicine Budapest. https://huveta.hu/bitstream/handle/10832/1745/Thesis%20BandW.pdf?sequence=1&isAllowed=y. Accessed 2025/05/31.
[66] X.Q. Shao, X.J. Yan, G.L. Luo, H.L. Zhang, X.L. Chai, F.X. Wang, J.K. Wang, J.J. Zhao, W. Wu, S.P. Cheng, Genetic evidence for domestic raccoon dog rabies caused by Arctic-like rabies virus in Inner Mongolia, China, Epidemiol. Infect. 139 (2011) 629–635. https://doi.org/10.1017/s0950268810001263.
[67] P. Sharma, C.K. Singh, D. Narang, Comparison of immunochromatographic diagnostic test with Hheminested Reverse transcriptase polymerase chain reaction for detection of rabies virus from brain samples of various species, Vet. World 8 (2015) 135. https://doi.org/10.14202/vetworld.2015.135-138.
[68] S.K. Singh, P.R. Islam, T. Hasan, The Prevalence of Clinical Diseases in Dogs of Sylhet Sadar, Bangladesh, Int. J. Pure Appl. Sci. Technol. 5 (2015) 41–45.
[69] S. Th, R. Wallace, T. Wj, W. Hy, S. Inoue, J. Liu, C. Weng, C. Fei, The Progression of Taiwan Ferret Badger Rabies from July 2013 to December 2016, J. Zoonotic Dis. Public Health 1 (2017) 1–6.

[70] W. Thanapongtharm, S. Suwanpakdee, A. Chumkaeo, M. Gilbert, A. Wiratsudakul, Current characteristics of animal rabies cases in Thailand and relevant risk factors identified by a spatial modeling approach, PLoS Neglect. Trop. Dis. 15 (2021) 1–16. https://doi.org/10.1371/journal.pntd.0009980.
[71] K.J. Tsai, W.C. Hsu, W.C. Chuang, J.C. Chang, Y.C. Tu, H.J. Tsai, H.F. Liu, F.I. Wang, S.H. Lee, Emergence of a sylvatic enzootic formosan ferret badger-associated rabies in Taiwan and the geographical separation of two phylogenetic groups of rabies viruses, Vet. Microbiol. 182 (2016) 28–34. https://doi.org/10.1016/j.vetmic.2015.10.030.
[72] B. Tuvshintulga, E. Batmagnai, E. Bazarragchaa, P. Dulam, S. Sugar, B. Battsetseg, Detection and molecular characterization of rabies virus in Mongolia during 2008-2010, Int. J. One Health 1 (2015) 26–31. https://doi.org/10.14202/IJOH.2015.26-31.
[73] J. Ubeyratne, L. Srikitjakarn, D. Pfeiffer, L. Kohnle, N. Sunil-Chandra, W. Chaisowwong, P. Hemwan, Canine Rabies and its Implications for Human Health in Sri Lanka, J. Vet. Sci. 4 (2018) 1–10.
[74] A. Vos, H. Ün, K. Hampson, K. De Balogh, O. Aylan, C.M. Freuling, T. Müller, A.R. Fooks, N. Johnson, Bovine rabies in Turkey: patterns of infection and implications for costs and control, Epidemiol. Infect. 142 (2014) 1925–1933. https://doi.org/10.1017/s0950268813002811.
[75] A.H. Vu, T.T. Nguyen, D.V. Nguyen, G.C. Ngo, T.Q. Pham, S. Inoue, A. Nishizono, T.D. Nguyen, A.K.T. Nguyen, Rabies-infected dogs at slaughterhouses: A potential risk of rabies transmission via dog trading and butchering activities in Vietnam, Zoonoses Public Health 68 (2021) 630–637. https://doi.org/10.1111/zph.12851.
[76] X. Wang, S. Chen, Y. Lei, K. Chen, Y. Zhang, Q. Tang, Complete genome sequences of two rabies virus isolates indicate Chinese ferret badger as a new natural inherent host, Journal of Applied Virology 2 (2013) 8–20.
[77] E. Wera, A.G.J. Velthuis, M. Geong, H. Hogeveen, Costs of rabies control: An economic calculation method applied to Flores Island, PLoS ONE 8 (2013). https://doi.org/10.1371/journal.pone.0083654.
[78] H. Wilde, T. Hemachudha, S. Wacharapluesadee, B. Lumlertdacha, V. Tepsumethanon, Rabies in Asia: the classical zoonosis, Curr. Top. Microbiol. Immunol. 365 (2013) 185–203. https://doi.org/10.1007/82_2012_228.
[79] Y. Liu, S. Zhang, X. Wu, J. Zhao, Y. Hou, F. Zhang, A. Velasco-Villa, C.E. Rupprecht, R. Hu, Ferret badger rabies origin and its revisited importance as potential source of rabies transmission in Southeast China, BMC Infectious Diseases 10 (2010) 1–7. https://doi.org/10.1186/1471-2334-10-234.
[80] Y. Liu, S. Zhang, J. Zhao, F. Zhang, R. Hu, Isolation of Irkut Virus from a Murina leucogaster Bat in China, PLoS Neglect. Trop. Dis, 7 (2013) 3–8. https://doi.org/10.1371/journal.pntd.0002097.
[81] G. Yale, A.D. Gibson, R.S. Mani, H. PK, N.C. Costa, J. Corfmat, I. Otter, N. Otter, I.G. Handel, B.M. Bronsvoort, Evaluation of an immunochromatographic assay as a canine rabies surveillance tool in Goa, India, Viruses 11 (2019) 649. https://doi.org/10.3390/v11070649.
[82] J. Yu, H. Li, Q. Tang, X. Tao, H. Wu, Z. Mo, H. Zhang, D. Wang, J. Weng, R. Shen, F. Zhu, X. Wang, H. Liu, X. Shen, S. Wang, [Study on the status of infection and distribution of rabies virus in China], Zhonghua liu xing bing xue za zhi = Zhonghua liuxingbingxue zazhi 31 (2010) 521–524.
[83] S. Zeynalova, M. Shikhiyev, T. Aliyeva, R. Ismayilova, E. Wise, R. Abdullayev, K. Asadov, S. Rustamova, F. Quliyev, A.M. Whatmore, E.S. Marshall, A.R. Fooks, D.L.

Horton, Epidemiological Characteristics of Human and Animal Rabies in Azerbaijan, Zoonoses Public Health 62 (2015) 111–118. https://doi.org/10.1111/zph.12119.
[84] S. Zhang, J. Zhao, Y. Liu, A.R. Fooks, F. Zhang, R. Hu, Characterization of a rabies virus isolate from a ferret badger (Melogale moschata) with unique molecular differences in glycoprotein antigenic site III, Virus Res. 149 (2010) 143–151. https://doi.org/10.1016/j.virusres.2010.01.010.
[85] S. Zhang, Y. Liu, Y. Hou, J. Zhao, F. Zhang, Y. Wang, R. Hu, Epidemic and maintenance of rabies in chinese ferret badgers (Melogale moschata) indicated by epidemiology and the molecular signatures of rabies viruses, Virol. Sin. 28 (2013) 146–151. https://doi.org/10.1007/s12250-013-3316-7.
[86] J.Y. ZHANG, B. ZHANG, S.F. ZHANG, F. ZHANG, N. LI, Y. LIU, R.L. HU, Dog-transmitted Rabies in Beijing, China, Biomed. Environ. Sci. 30 (2017) 526–529. https://doi.org/10.3967/bes2017.069.
[87] Y. Zhu, G. Zhang, M. Shao, Y. Lei, Y. Jiang, C. Tu, An outbreak of sheep rabies in Shanxi province, China, Epidemiol. Infect. 139 (2011) 1453–1456. https://doi.org/10.1017/s0950268811001348.
[88] N. Zhang, C. Song, X. Tao, W. Zhu, Epidemiologic Features of Human Rabies in China from 2015-2021, Zoonoses 3 (2023). https://doi.org/10.15212/ZOONOSES-2023-0012.
[89] R.T. Abdulmoghni, A.H. Al-Ward, K.A. Al-Moayed, M.A. Al-Amad, Y.S. Khader, Incidence, trend, and mortality of human exposure to rabies in Yemen, 2011-2017: Observational study, JMIR Public Health Surveill. 7 (2021) e27623. https://doi.org/10.2196/27623.
[90] A.J. Alyasiri, N.M. Mousa, A Comparative Study of Microbial & Parasitic , Disease in Al Muthanna Province , Iraq, Ann. Romanian Soc. Cell Biol. 25 (2021) 4972–4983.
[91] 1 Hai-Lin Zhang, J.-C.D. Yu-Zhen Zhang, 1 Wei-Hong Yang, 1 Xiao-Yan Tao, Hao Li, and Q.T. Yun Feng, Du-Juan Yang, Juan Zhang, Jiang He, Xin-Xin Shen, Li-Hua Wang, Yun-Zhi Zhang, Miao Song, Yunnan, Molecular Epidemiology of Reemergent Rabies in Yunnan Province, Southwestern China, Microbiol. Immunol. 51 (2014) 833–840. https://doi.org/10.1111/j.1348-0421.2007.tb03979.x.
[92] H. Li, J.J. Liu, S.J. Ding, L. Cai, Y. Feng, P.C. Yu, S.Q. Liu, X.X. Lu, X.Y. Tao, W.Y. Zhu, Human rabies in China: Evidence-based suggestions for improved case detection and data gathering, Infect. Dis. Poverty 9 (2020) 4–9. https://doi.org/10.1186/s40249-020-00672-9.
[93] R.S. Mani, A.M. Anand, S.N. Madhusudana, Human rabies in India: An audit from a rabies diagnostic laboratory, Trop. Med. Intern. Health 21 (2016) 556–563. https://doi.org/10.1111/tmi.12669.
[94] T. Matsumoto, K. Ahmed, D. Karunanayake, O. Wimalaratne, S. Nanayakkara, D. Perera, Y. Kobayashi, A. Nishizono, Molecular epidemiology of human rabies viruses in Sri Lanka, Infect. Genet. Evol. 18 (2013) 160–167. https://doi.org/10.1016/j.meegid.2013.05.018.
[95] A.K.T. Nguyen, D. V. Nguyen, G.C. Ngo, T.T. Nguyen, S. Inoue, A. Yamada, X.K. Dinh, D. V. Nguyen, T.X. Phan, B.Q. Pham, H.T. Nguyen, H.T.H. Nguyen, Molecular epidemiology of rabies virus in Vietnam (2006-2009), Jpn. J. Infect. Dis, 64 (2011) 391–396. https://doi.org/10.7883/yoken.64.391.
[96] B.N.H. Sim, B.N.W. Liang, W.S. Ning, S. Viswanathan, A retrospective analysis of emerging rabies: A neglected tropical disease in sarawak, Malaysia, J. R. Coll. Physicians of Edinb. 51 (2021) 133–139. https://doi.org/10.4997/JRCPE.2021.207.
[97] W. Suraweera, S.K. Morris, R. Kumar, D.A. Warrell, M.J. Warrell, P. Jha, Deaths from Symptomatically Identifiable Furious Rabies in India: A Nationally

Representative Mortality Survey, PLoS Neglect. Trop. Dis. 6 (2012). https://doi.org/10.1371/journal.pntd.0001847.
[98] M.R. Shirzadi, J. Pourmozafari, M. Shamsipour, Status of Animal bite and rabies cases in northeast provinces of Iran during 2009-2010, J. Zoonoses 1 (2014).
[99] N.K. Tenzin, J. Dhand, J. Dorjee, M.P. Ward, Re-emergence of rabies in dogs and other domestic animals in eastern Bhutan, 2005–2007, Epidemiol. Infect. 139 (2010) 220–225. https://doi.org/10.1017/S0950268810001135.
[100] Tenzin, B. Sharma, N.K. Dhand, N. Timsina, M.P. Ward, Reemergence of rabies in Chhukha district, Bhutan, 2008, Emerg. Infect. Dis.16 (2010) 1925–1930. https://doi.org/10.3201/eid1612.100958.
[101] K. Santhia, W. Sudiasa, Human rabies epidemiology in Bali, Indonesia, Intern. J. Health Med. Sci. 2 (2019) 7–16. https://doi.org/10.31295/ijhms.v2n1.77.
[102] B.N.H. Sim, B.N.W. Liang, W.S. Ning, S. Viswanathan, A retrospective analysis of emerging rabies: a neglected tropical disease in Sarawak, Malaysia, J. R. Coll. Physicians Edinb. 51 (2021) 133–139. https://doi.org/10.4997/jrcpe.2021.207.
[103] A.A. Al-Kubati, F.A. Badi, H.A. Golah, S.M.A. Al Maswari, Spatial-Temporal Distribution and Some Associated Factors of Rabies in Dhamar Governorate, Yemen: A Retrospective Study 2011–2017, Yemeni J. Agricult. Vet. Sci. 4 (2023). https://doi.org/10.70022/yjavs.v4i1.1762.
